# Number and Function of Bone-Marrow Derived Angiogenic Cells and Coronary Flow Reserve in Women without Obstructive Coronary Artery Disease: A Substudy of the NHLBI-Sponsored Women's Ischemia Syndrome Evaluation (WISE)

**DOI:** 10.1371/journal.pone.0081595

**Published:** 2013-12-02

**Authors:** Rajesh Mohandas, Larysa Sautina, Shiyu Li, Xuerong Wen, Tianyao Huo, Eileen Handberg, Yueh-Yun Chi, C. Noel Bairey Merz, Carl J. Pepine, Mark S. Segal

**Affiliations:** 1 Division of Nephrology, Hypertension & Transplantation, University of Florida, Gainesville, Florida, United States of America; 2 Division of Cardiovascular Medicine, University of Florida, Gainesville, Florida, United States of America; 3 Department of Biostatistics, University of Florida, Gainesville, Florida, United States of America; 4 Barbra Streisand Women's Heart Center, Cedars-Sinai Medical Center, Los Angeles, California, United States of America; National Cancer Institute, United States of America

## Abstract

**Background:**

In women with ischemia and no obstructive coronary artery disease, the Women's Ischemic Syndrome Evaluation (WISE) observed that microvascular coronary dysfunction (MCD) is the best independent predictor of adverse cardiovascular events. Since coronary microvascular tone is regulated in part by endothelium, we hypothesized that circulating endothelial cells (CEC), which reflect endothelial injury, and the number and function of bone-marrow derived angiogenic cells (BMDAC), which could help repair damaged endothelium, may serve as biomarkers for decreased coronary flow reserve (CFR) and MCD.

**Methods:**

We studied 32 women from the WISE cohort. CFR measurements in response to intracoronary adenosine were taken as an index of MCD. We enumerated BMDAC colonies and CEC in peripheral blood samples. BMDAC function was assessed by assay of migration of CD34+ cells toward SDF-1 and measurement of bioavailable nitric oxide (NO). These findings were compared with a healthy reference group and also entered into a multivariable model with CFR as the dependent variable.

**Results:**

Compared with a healthy reference group, women with MCD had lower numbers of BMDAC colonies [16 (0, 81) vs. 24 (14, 88); P = 0.01] and NO [936 (156, 1875) vs. 1168 (668, 1823); P = 0.02]. Multivariable regression analysis showed strong correlation of CFR to the combination of BMDAC colony count and CD34+ cell function (migration and NO) (R^2^ = 0.45; P<0.05).

**Conclusions:**

The BMDAC function and numbers of BMDAC colonies are decreased in symptomatic women with MCD and are independently associated with CFR. These circulating cells may provide mechanistic insights into MCD in women with ischemia.

## Introduction

Ischemic heart disease (IHD) remains the leading cause of death and a major cause of disability among women. However, the majority of women with symptoms and other findings suggesting ischemia referred to coronary angiography have no severe coronary obstruction [1], [2]. Standard risk stratification schemes, developed mainly for patients with obstructive coronary artery disease (CAD), underestimate risk for cardiovascular-related adverse outcomes among women [3]. The Women's Ischemia Syndrome Evaluation (WISE) was initiated by the National Heart, Lung and Blood Institute (NHLBI) to improve our understanding of pathological mechanisms and diagnostic evaluation of IHD in women [4]. Among such women with ischemia but no obstruction on coronary angiograms, microvascular coronary dysfunction (MCD), as evidenced by decreased coronary flow reserve (CFR) to intracoronary adenosine, was the best independent predictor of major cardiovascular events over 5.4 years of follow- up [5]. However, clinical characteristics, atherosclerosis risk factors, functional capacity, and inflammatory markers, though linked with reduced CFR, account for less than 20% of variability in CFR in the WISE [6]. Determination of CFR is invasive and expensive, and there is interest in developing less invasive biomarkers to predict an individual's coronary microvascular physiology, response to treatment, and adverse outcomes.

Recently, circulating endothelial cells (CEC) [7]–[9] and bone-marrow derived angiogenic cells (BMDAC) [10]–[12] isolated from peripheral blood have been suggested as markers of vascular or endothelial health. Evolving data suggest that CEC are endothelial cells derived from the intima and express mature endothelial markers such as CD146 [13], [14]. CEC indicate vascular damage, and their numbers are elevated in the presence of endothelial damage [7], [9], [15]. Women with MCD have reduced flow-mediated vasodilatation and an abnormal response of coronary microcirculation to acetylcholine, suggesting that endothelial dysfunction might have a role in the pathogenesis of MCD [16]. BMDAC are cells that express progenitor cell markers (CD34 and/or CD133) and endothelial markers (vascular endothelial growth factor receptor 2) by flow cytometry, or, when cultured in the presence of media supplemented with endothelial growth factors, can differentiate into mature endothelial cells [10], [17], [18]. BMDAC mobilize and home to sites of ischemic injury and accelerate neovascularization [19]. The number of circulating BMDAC appears to be an independent predictor of subclinical atherosclerosis [20], [21] and of future cardiovascular events [22]. In other patient populations, flow mediated dilatation is closely related to the number of BMDAC [11], supporting their possible role in MCD. Porto et al have demonstrated that BMDAC respond to ischemia and not myocardial necrosis, raising the possibility that BMDAC may be stimulated in women with MCD [23]. We have previously shown that BMDAC isolated from diabetic patients have cytoskeletal alterations preventing responsiveness to growth factor stimulation, which can be corrected by use of exogenous nitric oxide (NO) [24]. Interestingly, L-arginine, which increases NO levels, improved endothelial dysfunction in women with MCD [16]. In addition to the absolute numbers, the ability of BMDAC to mobilize in response to ischemia and growth factors may affect the reparative process in response to endothelial injury.

We hypothesized that the number of CEC, which reflect endothelial injury, and the number and function of circulating BMDAC, which potentially participate in repair of injured endothelium, might serve as noninvasive biomarkers for MCD.

## Methods

### Ethics statement

All study participants gave written informed consent before undergoing evaluation. These studies conform to the ethical principles outlined in the 1975 Declaration of Helsinki and were approved by the University of Florida Institutional Review Board.

### Study protocol and study population

The Women's Ischemia Syndrome Evaluation (WISE) (study protocol registry: http://www.clinicaltrials.gov/ct2/show/NCT00000554) is an NHLBI-sponsored study aimed at improving diagnostic evaluation and understanding pathological mechanisms of IHD in women. The design was a prospective cohort, and protocol details, including selection criteria, have been previously published in detail [4]. Briefly, clinically stable women with angina and other findings suggesting ischemia who were referred for coronary angiography were enrolled in the WISE cohort at the University of Florida. Consecutive women who had no obstructive CAD and had undergone coronary reactivity testing were recruited for this ancillary study. Absence of obstructive CAD was defined as <50% luminal obstruction in any epicardial coronary artery on angiography. A reference population included women recruited from the community, matched for age, body mass index (BMI) and free of any chronic illnesses such as diabetes, hypertension, or other cardiovascular disease. Demographic data were recorded on standardized WISE questionnaires and transmitted to the WISE Data Coordinating Center (University of Pittsburgh).

Baseline evaluation included physical examination and collection of clinical and laboratory data. Qualitative and quantitative coronary angiographic analyses were done by the WISE Angiographic Core Laboratory (Brown University) masked to patient data; detailed methodology has been published [25]. Presence of any ≥50% diameter stenosis was defined as obstructive CAD, 20% to 49% as mild CAD, and <20% as no CAD. In addition, a CAD severity score was assigned based on the aggregate of percent stenosis, extent and location of stenosis, and degree of collateral vessels, as previously described [25]. For borderline lesions, at the discretion of the interventionist, intravascular ultrasound or fractional flow reserve was used to confirm absence of obstructive stenosis.

### Coronary reactivity testing protocol

Coronary reactivity testing was performed in a stenosis free area of the left anterior descending coronary artery when possible, with the left circumflex artery as a secondary choice. A Doppler-tipped guidewire (0.014-inch FloWire, JOMED/Cardiometrics, Mountain View, CA, now Volcano Corporation, San Diego, CA) was advanced through the diagnostic catheter, and when a stable velocity signal was obtained, baseline recordings were made. Intracoronary bolus injections of 18 mcg of adenosine (Adenocard, Fujisawa USA, Deerfield, IL), a predominantly non–endothelium-dependent microvascular dilator, were administered into the left main coronary artery. At least 3 injections were done to assure a stable average peak blood flow velocity was obtained, with return to baseline velocity documented before each bolus. Pulsed-wave Doppler flow spectra were used to calculate time-averaged peak velocity (APV). Recordings were analyzed at the WISE CFR Core Lab (University of Florida) masked to all other data, and CFR was defined as the ratio of APV after adenosine to average baseline velocity just before adenosine. In prior WISE studies, this measure correlated closely (r = 0.87, P<0.001) with volumetric flow [26].

### Isolation of CD34+ BMDAC

A minimum of 8 ml of peripheral blood collected in CPT tubes with heparin (BD Biosciences, Franklin Lakes, NJ) was centrifuged. The mononuclear cells were pelleted, washed twice and then resuspended in 100 µl PBS supplemented with 2 mmol/L EDTA, to which 33 µl of FcR-blocking reagent and 33 µl of magnetic microbeads conjugated with an anti-CD34 antibody (Miltenyi Biotec, Auburn, CA) were added. After incubation for 30 min at 4°C, the cells were diluted in 10× volume of PBS supplemented with 2 mmol/L EDTA supplemented with 0.1% BSA. The CD34+ cells were positively selected by automated magnetic selection (autoMACS, Miltenyi Biotec). Selected cells were confirmed to be CD34+ cells staining with phycoerythrin-conjugated anti-CD34 (Miltenyi Biotec) and flow cytometry.

### Measurement of BMDAC intracellular NO

The BMDAC were incubated with 5 µM 4-amino-5-methylamino-2,7-difluorofluorescein (DAF-FM) diacetate for 30 min at 37°C in the dark. Excess extracellular probe was removed by washing in Hanks balanced salt solution followed by incubation for 10 min at room temperature to allow for probe de-esterification. Green fluorescence was measured using an inverted microscope Axiovert 200 (Carl Zeiss) equipped with CCD camera and image acquisition/analysis software AxioVision (Version 4.5). Fluorescence intensity was measured in 20 to 30 cells per field in at 3–4 fields per experiment.

### Migration of BMDAC

Chemotaxis was assessed by staining the CD34+ cells with CellVue Burgundy (C1004, Molecular Targeting Technologies) before loading onto a Boyden Chamber. SDF-1 was loaded in the bottom chamber onto which a polycarbonate membrane (8 µm pores; Neuro Probe, Gaithersburg, MD) coated with 10% bovine collagen was overlaid and the cells were loaded in the top chamber. After 4 h at 5% CO2 at 37°C, the percentage of cells that migrated was determined by collecting media in the lower chamber and determining relative fluorescence using Odyssey® Infrared Imaging System (LI-COR Biosciences, Lincoln, NE) at excitation of 683 nm and an emission of 707 nm. Percentage increase in migration was calculated as maximum increase in migration in response to SDF as compared to baseline (no SDF).

### Culture of BMDAC

Peripheral blood mononuclear cells were isolated from 8 ml of peripheral blood collected in CPT tubes (Becton Dickinson, San Jose, CA). Cells were enumerated, resuspended in Endocult medium (Stem-Cell Technologies, Seattle, WA), and cultured on 6-well plates precoated with human fibronectin (Becton Dickinson). After 48 hours, new medium was added, and nonadherent cells were transferred to a new fibronectin-coated plate to remove monocytes and mature endothelial cells. After 3 days of culture, BMDAC colony–forming units (CFU) were enumerated by light microscopy.

### Enumeration of CEC

The mononuclear cell fraction from the CPT tubes was incubated with immunomagnetic beads conjugated with P1H12, a murine, monoclonal antibody specific for human endothelial cells. The rosetted cells were cytospun onto poly-L-lysine coated slides. After drying overnight, slides were fixed with 1% paraformaldehyde and stained with propidium iodine in phosphate-buffered saline (PBS), prior to mounting the slides in Vectashield with DAPI (Vector Laboratories, Inc., Burlingame, CA). Quantitation of CEC was performed by identification of the cells using a Zeiss Axiophot microscope (CarlZeiss, Inc., Thornwood, NY) and expressed as number per ml of blood.

### Statistical Methods

The statistical analyses were conducted in SAS 9.3 (SAS Institute, Cary, NC). Clinical characteristics were presented as either mean ± SD for continuous variables or % for categorical variables. Number of BMDAC colonies, NO, and migration were expressed as median (range), and transformed to log base _2_ to satisfy the assumptions of homogeneity, linearity, and Gaussian distribution of errors. The data were transformed as follows: log_2_CEC = log_2_(CEC/10); log_2_Migration = log_2_(% increase in migration/10+0.01); log_2_NO = log_2_(NO/100); log_2_ BMDAC  = log_2_(BMDAC/10+0.01). We compared the log transformed BMDAC number and function between the WISE cohort and healthy reference group using the Satterthwaite two-sided t-test. Multivariable linear regression analysis was conducted with CFR as the dependent variable. Pearson's correlation between variables and condition index of each variable were checked using a linear regression model to detect collinearity. Cell covariates include BMDAC colony number, maximum % increase in migration of BMDAC to SDF, NO in BMDAC, CEC, with clinical covariates including diabetes, hypertension, hyperlipidemia, age and double product of HR×SBP. A backward model selection was employed, and CEC, diabetes, hypertension and hyperlipidemia were dropped sequentially from the model because of their insignificant effects. Age, maximum % increase in migration of BMDAC to SDF, and NO in BMDAC were retained in the model to improve overall model fit. A P-value <0.05 was considered statistically significant.

## Results

Pertinent baseline characteristics of the WISE women studied are summarized in Table 1. The majority was Caucasian, middle-aged and obese; smokers and diabetics constituted a minority. Almost half (41%) had hypertension. The reference group, as selected, was similar in age and BMI but without clinical cardiovascular disease, diabetes, hypertension, or other known chronic illness. Triglyceride levels were lower in the reference group vs. the WISE women (92 vs.164).

**Table 1:** Pertinent characteristics of the study populations.

Clinical Characteristics		Healthy Reference Group (n = 11)	WISE Cohort (n = 32)	P-value
Race White, %		64	88	0.17
Age (years) (mean SD [range])		43 (12 [30–63])	50 (9 [36–73])	0.06
BMI (mean SD [range])		28 (5 [21–38])	31 (8 [19–47])	0.32
Cholesterol (mg/dl) (mean SD [range])		183 (31 [142–230])	200 (40 [122–263])	0.13
Triglycerides (mg/dl) (mean SD [range])		92 (59 [48–253])	164 (99 [24–385])	0.04
History of hypertension, %		0	41	-
History of diabetes, %		0	18	-
History of smoking, %		36	32	>0.99
Menopausal, %		18	70	0.004
HR * SBP ÷1000 (mean SD [range])		8.8 (1.6 [7.4–12.5])	9.0 (1.9 [5.0–12.9])	0.80
DBP (mmHg) (mean SD [range])		76 (9 [57–88])	71 (11 [49–88])	0.14
DASI score (mean SD [range])		—	24 (20 [0–58])	
Coronary artery disease (CAD)				
	(0–20% stenosis), %		73	
	(20–40% stenosis), %		23	
	(>40% stenosis), %		3	
CAD severity score (mean SD [range])		-	5.98 (2.08 [5–13.75])	
Medications				
	Lipid Lowering Agents, %	—	15	
	Statins, %	—	30	
	Nitrates, %	—	27	
	Calcium channel blockers, %	—	6	
	Beta blockers, %	—	36	
	Antiplatelet agents, %	—	3	
	ACE inhibitors, %	—	74	
	Angiotensin receptor blockers, %	—	6	
CFR		—	2.75 (0.68)	
Baseline APV (cm/s) (SD)			24.1 (8.5)	
Peak APV post adenosine (cm/s) (SD)		—	62.4 (12.1)	

SD  =  standard deviation; BMI =  body mass index; HR =  heart rate; SBP =  systolic blood pressure; DBP =  diastolic blood pressure; DASI =  Duke Activity Status Index; CFR =  coronary flow reserve; APV =  average peak velocity.

### Women with symptoms/signs of ischemia and no obstructive CAD by coronary angiogram have reduced BMDAC colonies and function

**Figure 1:**
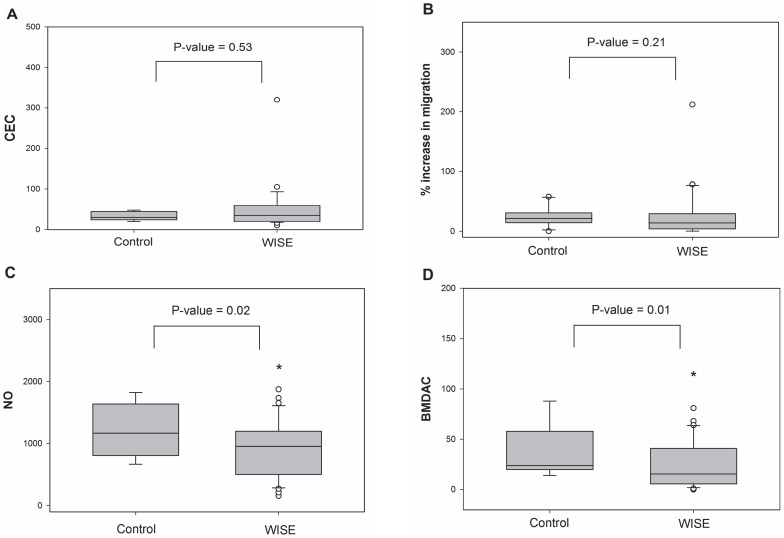
Bone-marrow derived angiogenic cell (BMDAC) colonies and intracellular nitric oxide (NO) are reduced in the WISE cohort compared to healthy reference group. (**A** and **B**) Number of circulating endothelial cells and migration ability of CD34+ cells to SDF-1 in the WISE cohort and healthy reference group. (**C**) Intracellular NO measurement in BMDACs in the WISE cohort and healthy reference group. (**D**) BMDAC colonies in the WISE cohort and healthy reference group. Horizontal bars indicate the median; upper and lower edges of box are 75th and 25th percentiles. *P<0.05.

Compared with the age, BMI, and sex-matched healthy reference group, the WISE cohort had fewer BMDAC colonies [16 (0, 81) vs. 24 (14, 88); P = 0.01]. The intracellular NO in BMDAC was lower in the WISE cohort than in the healthy reference group [936 (156, 1875) vs. 1168 (668, 1823); P = 0.02]. The BMDAC function, as measured by migration toward SDF, was not significantly different between the WISE cohort and the healthy reference group [14 (0, 212) vs. 21 (0, 58); P = 0.21]. The box plots comparing the cell variables and the results of the t-test comparing the WISE cohort to the healthy reference group are shown in Figure 1.

### The number and function of BMDAC correlate with coronary flow reserve (CFR)

**Figure 2:**
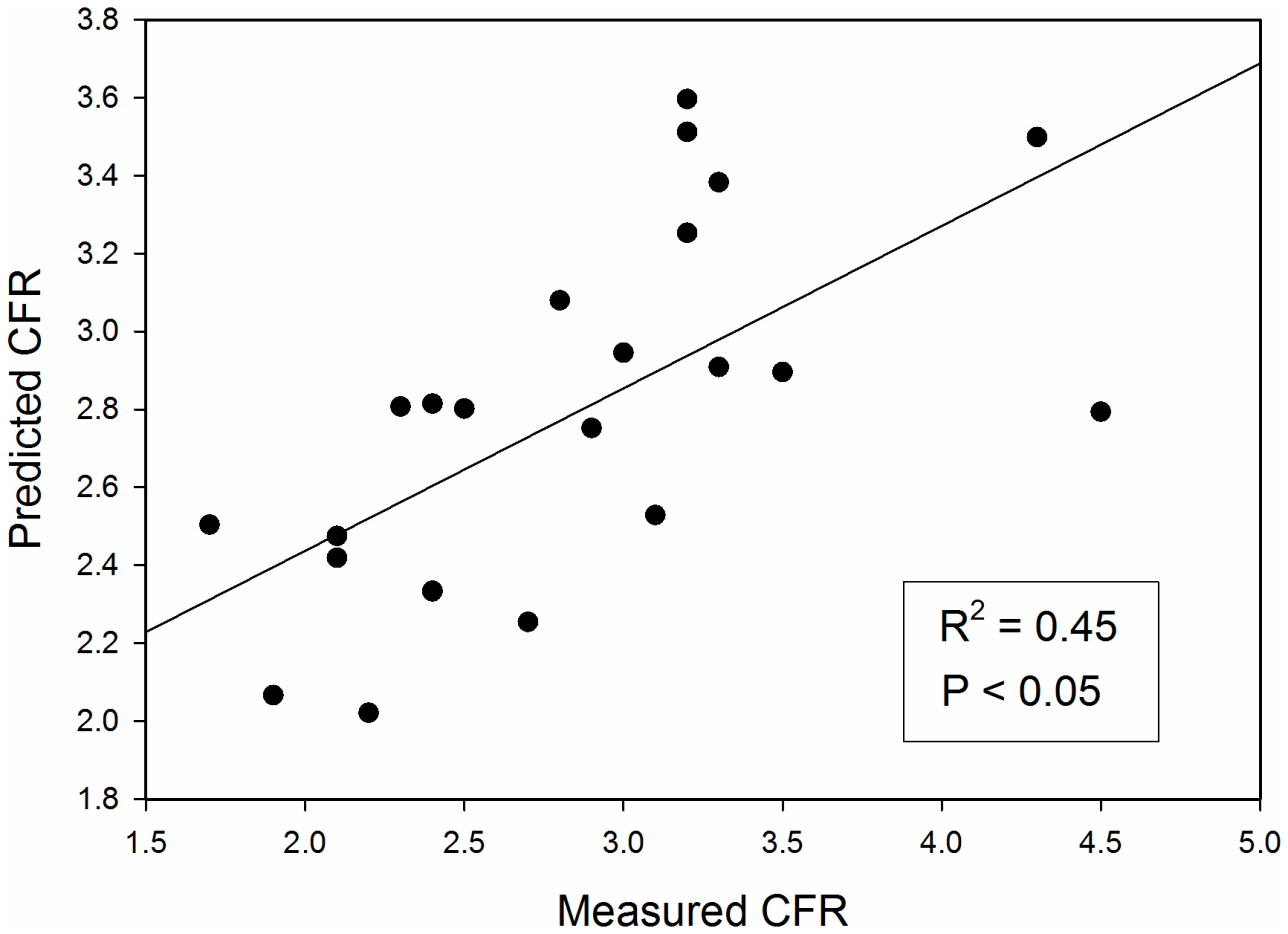
The coronary flow reserve (CFR) predicted from the cell variables closely approximates the measured CFR (R^2^ = 0.45, P<0.05).

A total of 23 patients with complete data were included in the regression analysis; patients with incomplete data were excluded. Univariate analysis showed a significant correlation between Log_2_CFR and Log_2_BMDAC (r = 0.50 p = 0.02). Traditional risk factors for atherosclerosis such as diabetes, hypertension, and hyperlipidemia did not show a significant correlation to CFR. Although BMDAC function by itself was not associated with CFR, in multivariable regression the combination of BMDAC colonies and function significantly correlated with CFR (R^2^ = 0.45, *P*<0.05), when controlled for cardiac work (HR×SBP) and age. The model is summarized in Table 2. The CFR predicted by the model closely approximated the measured CFR; the plot and predicted CFR are shown in Figure 2.

**Table 2:** Multivariate analysis between patient characteristics, circulating cell characteristics, and coronary flow reserve.

Parameter*	Beta	Standard Error	t-value	P-value
Age	0.04	0.08	0.53	0.60
HR×SBP	−0.07	0.03	−2.18	0.04
Log_2_BMDAC	0.11	0.04	2.70	0.015
Log_2_SDF	−0.02	0.02	−0.95	0.35
Log_2_NO	−0.07	0.07	−0.92	0.37

* For numerical purposes, age was divided by 10 and HR×SBP was divided by 1000 when used to fit the regression model.

HR =  heart rate; SBP =  systolic blood pressure; BMDAC =  bone-marrow derived angiogenic cells; NO =  nitric oxide.

### CEC are not increased in the WISE cohort

There was no difference in the number of CEC between the WISE group and healthy reference group [35 (10, 320) vs. 30 (20, 48); P = 0.53]. We did not observe a significant contribution of CEC to CFR.

## Discussion

### BMDAC number and function in CAD

Recent studies indicate that the number and functionality of BMDAC may predict clinical outcomes in patients with CAD. Vasa et al reported that number and migratory capacity of BMDAC (as CD34+KDR+ cells) are impaired in patients with CAD compared with age-matched healthy controls [27]. Furthermore, even among subjects with various degrees of risk but no history of cardiovascular disease, the number of BMDAC colonies was a better predictor of endothelial dysfunction than the Framingham risk score [11]. Turan et al found the number of BMDAC (CD133+ CD45+ cells) decreased as CAD severity (SYNTAX score) increased [28]. Consistent with these findings, Werner et al found that increased levels of BMDAC (as CD34+KDR+ cells) were associated with decreased risk of adverse outcomes [29]. Thus, in patients with or at risk for CAD, the available data indicate that the number and function of BMDAC are closely linked with adverse outcomes.

### BMDAC number and function in MCD

We are aware of only two published reports among patients with suspected MCD dealing with BMDAC number and function: both are relatively small in sample size with few women and coronary microvascular function was not directly measured. Shmilovich et al [30] examined number and functional properties of circulating BMDAC from 17 patients with suspected MCD (12 women) and 20 subjects in the reference group (14 women) matched for age, risk factors, and medications. They observed that patients with suspected MCD had increased numbers of circulating BMDAC (both CD34+/KDR and CD34+/CD133+ cells) vs. the reference group. The BMDAC proliferative capacity, as measured by BMDAC colonies and ability to support in vitro tube formation, were impaired in suspected MCD patients vs. the reference group. However, adhesiveness of BMDAC from suspected MCD patients to fibronectin and cultured mature endothelial cells was enhanced compared with the reference group. The authors concluded that suspected MCD patients have an altered circulating BMDAC phenotype that could potentially aid in understanding the pathogenesis of the syndrome. In the other report, Huang et al [31] measured the number and adhesive function of BMDAC in peripheral blood samples from patients with suspected MCD [n = 12 (6 women)], stable CAD [n = 12 (5 women)], and healthy controls [n = 10 (6 women)]. Reduced numbers of CFU of BMDAC were noted in both the suspected MCD and CAD patients vs. controls, and BMDAC number was associated with reduced peripheral artery flow-mediated dilatation (FMD) and low HDL cholesterol level. However, unlike Shmilovich et al, the authors found attenuated fibronectin adhesion function of BMDAC in patients with suspected MCD and CAD vs. controls. The authors suggested that their work “clearly showed for the first time” that, compared with normal individuals, patients with suspected MCD have decreased numbers and adhesive function of circulating BMDAC. They speculated that these findings may explain underlying mechanisms contributing to endothelial dysfunction and MCD.

Both studies relied on angina, evidence of ischemia on clinical non-invasive testing, and absence of obstructive CAD on a clinical coronary angiogram for a diagnosis of suspected MCD, and they also included very few women (12 and 6, respectively). Our study included 32 women, a prospectively defined protocol which required that angiograms and CFR recordings be read by core labs masked to other patient data, and determination of MCD by direct intracoronary blood flow measurements in response to adenosine to define CFR. We found that the ability of BMDAC to form colonies was not only markedly reduced in the WISE cohort, but the number and function of these cells were significantly associated with measured CFR.

Although traditional risk factors such as diabetes, hypertension, and hyperlipidemia are associated with decreased BMDAC number and function, consistent with our previously published work in a larger cohort of patients [6], they did not independently contribute significantly to CFR. However, removing diabetes and hypertension improved the final model, suggesting that although independently they have no effect on CFR, in concert they might reduce CFR, possibly by affecting BMDAC.

We did not find any difference in migration between WISE and healthy women. The production of NO in CD34 cells was reduced in the WISE cohort (2.92, SD = 0.92, vs. 3.51, SD = 0.53; P = 0.02). NO is thought to be a key mediator of BMDAC dysfunction in diabetes [24]. Interestingly, Bellamy et al. showed that L-arginine restored FMD and exercise capacity in women with MCD [16]. Thus, the low NO in CD34+ BMDAC could have a pathogenetic role in MCD. However, we did not see a significant contribution of intracellular NO to CFR. NO can be derived from different NO synthase (NOS) isoforms, the constitutively expressed endothelial NOS (eNOS), inducible NOS (iNOS), and neuronal NOS (nNOS). iNOS is upregulated immediately after myocardial infarction, and inhibition of iNOS has been shown to limit infarct size [32]. Elucidating the isoforms of NOS might help to further clarify the relationship of intracellular NO levels and CFR.

Surprisingly, the numbers of circulating CEC, a marker of endothelial injury, were not significantly different between the WISE cohort and healthy reference group. However the role of endothelial injury in MCD is not entirely clear. Bellamy and colleagues, as well as others, have shown that patients with Syndrome X have reduced flow-mediated vasodilatation, a surrogate marker for endothelial dysfunction [16]. Markers of endothelial activation such as ICAM, VCAM [33] and vWF [34] are also increased in patients with MCD. Quyyumi et al. showed that, in women with chest pain and normal coronary angiograms, the response of coronary arteries to acetylcholine correlated with decreased coronary vasodilator response to tachycardia (atrial pacing), suggesting that endothelial dysfunction may contribute to a decrease in coronary vasodilatory response in these patients [35]. However, some patients in the group had a normal response to acetylcholine. In a similar cohort of 42 women with ischemia and no coronary obstruction followed long-term, Bugiardini et al showed that endothelial dysfunction predicts adverse cardiac events and development of atherosclerotic CAD [36]. Patients with MCD are a heterogeneous group, and whether endothelial dysfunction identifies a group of patients at risk for atherosclerotic disease or those with subclinical CAD, or has a key pathogenetic role in MCD, needs to be clarified.

### Study Limitations

The main limitation of our study was the small number of subjects, which limited subgroup analysis and the ability to assess more complex interactions between CFR and the determinant variables. Nevertheless, this is the largest number of women with directly measured MCD and cell number and function analyses yet to be reported. Since it would have been unethical to subject our reference group to coronary angiography, we chose a population that was free from known CAD and risk factors for atherosclerosis. This reference group, though closely matched to study subjects, was slightly younger (not statistically significant) and had lower triglyceride levels. Moreover, our study only included women, most of whom were postmenopausal. However, our results are consistent with the current understanding of BMDAC function. The model appears sensitive to changes in cardiac work load approximated by the double product (HR X SBP). The ability of cardiac work to predict CFR has been substantiated in previous studies [6] and contributes to the validity to our model. In addition, it is important to note that there is no standard marker of BMDAC and different methods have been used for identification and quantification of BMDAC, making comparisons across studies difficult. A new BMDAC colony assay identified a primitive endothelial progenitor cell (EPC) stage with highly proliferative activity and a definitive EPC stage with vasculogenic properties [37]. Thus the functional properties of BMDAC may be different depending on their developmental stage, and thus may vary among individual patients as well as the type and severity of CAD. Clearly, better methods of identifying BMDAC and large-scale studies are needed to better evaluate whether BMDAC numbers and functions predict microvascular dysfunction among women.

### Conclusion

In conclusion, the decreased number and function of BMDAC in the WISE cohort, the correlation of BMDAC number and function with CFR compared with the healthy reference group, and the independent association of number of BMDAC colonies with CFR suggest that these cells might have a role in the pathogenesis of MCD. These circulating cells provide mechanistic insights into the pathophysiology of MCD, and interventions designed to correct functional abnormalities of BMDAC might have therapeutic potential. Further studies are needed to confirm these findings in a larger cohort and to identify if these findings are applicable in other conditions with a reduced CFR.
